# Prisons and the COVID-19 pandemic

**DOI:** 10.1017/ipm.2020.65

**Published:** 2020-05-27

**Authors:** G. Gulati, C. P. Dunne, B. D. Kelly

**Affiliations:** Graduate Entry Medical School, University of Limerick, Limerick, Ireland, (Email: Gautam.gulati@hse.ie); Department of Psychiatry, Trinity College, Dublin, Ireland

The COVID-19 pandemic is one of the most significant public health challenges for a generation. There are over 10 million people in prisons worldwide. They represent a population with a significantly higher prevalence of physical and psychiatric morbidity and vulnerability to adverse outcomes such as suicide and premature death (Fazel & Danesh, 2002; Fazel *et al*. 2011; Wildeman & Wang, 2017; Gulati *et al*. 2019). A closed community such as a prison presents a unique infection control challenge for rapid viral spread particularly in light of prison overcrowding which is common in both developed and developing countries.

Prisoners retain a right to parity of medical care and a right to bodily integrity despite restrictions in their liberty. This is enshrined in guidance from the United Nations through the ‘Mandela rules’ (United Nations Office for Drugs and Crime, 2015).

Measures that seek to control the spread of COVID-19, such as quarantine, can be associated with anxiety and psychological distress (Brooks *et al.* 2020). This is likely to be further heightened in individuals, such as prisoners, who may perceive a particular sense of powerlessness in their situation. The current crisis has necessitated restrictions in prison visiting arrangements which may further heighten the feeling of isolation. This has led to adverse events. For example, 12 Italian prisoners died in a riot linked with restrictions owing to the pandemic (Euronews & AFP, 2020). A prison in the United Kingdom reported its first COVID-19 related death on 26th March 2020 (The Guardian, 2020) and by 28th April 2020, there were 2000 possible cases and 15 reported deaths in UK prisons (Shaw, 2020). Prison-based outbreaks of the virus have been reported in China with over 500 cases (Reuters, 2020) and the USA which reported 30 deaths and 1300 cases whilst also recording the death of a prisoner giving birth (BBC News, 2020).

It is important that prisons are not forgotten in the public health response to this crisis (Bedford *et al.* 2020). Established principles such as social distancing, early identification of cases, ‘cocooning’ of the most vulnerable and assertive treatment of those who become unwell will likely have similar benefits in prisons as in non-prison settings. A number of these measures have been enacted by the Irish Prison Service in consultation with the National Public Health Emergency Team (Department of Justice and Equality, 2020). As a result, there have been no reported outbreaks amongst inmates in Irish prisons in the first several weeks of the pandemic.

We recommend seven key considerations that may be applicable internationally:

1. Measures should be undertaken to reduce the prison population through alternative criminal justice disposals and facilitating early release of low-risk offenders (Simpson & Butler, 2020)
2. Prisons should move to non-shared accommodation, that is, facilitate single-cell accommodation with in-cell sanitation.
3. There should be systematic and robust screening of new prisoners on reception for a history of travel and symptoms of COVID-19.
4. Local arrangements should be made by prisons with a designated hospital for transfer and treatment of suspected or confirmed COVID-19 illness.
5. Robust arrangements for ongoing physical health and mental healthcare of non-COVID-19 illnesses should be maintained; such illnesses are often risk factors for adverse outcomes with COVID-19.
6. Brief psychological intervention and/or psychoeducation addressing the understandable anxiety faced by those worried about the illness both in respect of themselves and their family should be offered and undertaken (Kelly, 2020).
7. As a vulnerable population, prisoners should receive parity of access to novel treatments and vaccines as they become available.

It is important that prison healthcare staff are not redeployed out of prisons during the crisis: such decisions are prone to influence from value judgements about who may or may not be deserving of treatment. This includes decisions around the redeployment of staff from psychiatric prison in-reach services who, like mental health services in the community, are likely to play an essential role (Cullen *et al.* 2020) in helping their patients during this pandemic. If anything, this is the time to enhance care for the most vulnerable.
